# Taking hospital treatments home: a mixed methods case study looking at the barriers and success factors for home dialysis treatment and the influence of a target on uptake rates

**DOI:** 10.1186/s13012-015-0344-8

**Published:** 2015-10-27

**Authors:** Gill Combes, Kerry Allen, Kim Sein, Alan Girling, Richard Lilford

**Affiliations:** Institute of Applied Health Research, University of Birmingham, Birmingham, B15 2TT UK; Health Services Management Centre, University of Birmingham, Birmingham, B15 2TT UK; Hull York Medical School, University of Hull, Hull, HU6 7RX UK; Warwick Medical School, University of Warwick, Coventry, CV4 7AL UK

**Keywords:** Targets, Dialysis, Home treatment, Self-care, Barriers, Facilitators, Patient education, Emotional support

## Abstract

**Background:**

Despite healthcare policies and evidence which promote home dialysis, uptake rates have been falling for over 10 years in England. A target introduced by commissioners in the West Midlands provided a unique opportunity to study how hospitals can increase home-based treatment for a group of patients with complex life-threatening conditions.

**Methods:**

Quantitative changes in home treatment uptake rates in seven hospitals in the West Midlands were compared with the rest of England for 3 years pre and post the introduction of the target in 2010, using a logistic regression model. Qualitative interviews in four hospitals with 96 clinical and managerial staff and 93 dialysis patients explored the barriers and facilitators to increasing the uptake of home treatment and the impact of the target.

**Results:**

Home treatment uptake rates increased significantly in the seven study hospitals compared with the 3 years prior to the introduction of the target and compared with the rest of England where rates remained static. The four main factors facilitating increased uptake were as follows: the commissioner’s target, linked to financial penalties; additional funding for specialist staff and equipment; committed, visible clinical champions and good systems for patient training and ongoing healthcare support at home. The three main barriers were as follows: lack of training for non-specialist staff, poorly developed patient education and considerable unrecognised and unmet emotional and psychological patient needs.

**Conclusions:**

This study shows the impact of using targets with financial penalties to achieve change and how hospitals can increase significantly the uptake of home-based self-care for a group of patients with complex medical needs. It provides useful pointers to the main barriers and facilitators, which are likely to be relevant to other groups of patients who could be treated at home. It also highlights two neglected areas which need to improve if patients with life-threatening long-term conditions are to be encouraged to take up home treatment: individualised patient education which allows exploration of the impacts of treatment options and the provision of ongoing emotional support.

## Background

Policy in many parts of the world, including England, favours shifting services from hospital to community, with an emphasis on home-based care and self-care [1–5]. Numerous studies have shown that services can be transferred successfully from hospital to home on a localised basis, for example, hospital at home schemes for COPD patients [6] and cardiac failure services [7]. However, these successful localised projects tend not to have led to wider region or country roll-out. Patients with end-stage renal failure, who are on dialysis, are a group of patients with a complex condition where there is considerable potential to have large-scale shift from care provided by health professionals in hospital settings to self-care at home. Home-based treatments tend to be less burdensome and expensive than care in hospital, and therefore tend to be viewed favourably by patients and policymakers alike. As a result, national renal policies have promoted home dialysis for more than a decade [8–11], supported by favourable evidence for its clinical and cost effectiveness [12–14].

Despite this evidence and the favourable policy context, home dialysis uptake rates in England have been falling, declining by 42 % between 2002 and 2009 (from 30.7 to 17.8 % [15, 16]). It was against this background that the service commissioner for the West Midlands introduced a 5-year target in April 2010 for increasing to 35 % the proportion of dialysis patients on home treatments, with failure to meet annual interim targets resulting in a loss of up to 1 % of the total renal income per annum. At this time, the uptake of home dialysis in the West Midlands, covering a population of 5.6 million, was at 17.3 %, marginally below the England average of 17.8 % [16]. This provided an opportunity to study the impact of an imposed target plus financial penalty (known as pay-for-performance) and identify factors which might affect how far the target incentivised hospitals to increase the uptake of home dialysis. The literature on this topic is large, complex and of mixed quality [17, 18]; however, systematic review evidence does suggests that pay-for-performance is most effective when it operates at the level of a team/clinical service [19]. The most recent review of systematic reviews highlights that how a pay-for-performance scheme is designed and how it is implemented can both influence how effective it is at changing practice [18].

This study also provided an opportunity to examine more broadly the barriers and facilitators to increasing the uptake of home dialysis as an exemplar of the challenges involved in shifting from hospital-based care provided by health professionals to home-based self-care, for a group of patients with complex medical needs. The mixed methods enabled quantitative changes in uptake rates to be tracked over a 3-year period and used qualitative case studies to explore and explain how hospitals were achieving increases in the uptake of home-treatment and how the target was operating.

In this article, we use the term “In-centre haemodialysis” to mean haemodialysis provided by nurses in a hospital- or community-based dialysis unit. “Home dialysis” means peritoneal dialysis (PD) and home haemodialysis (HHD) which patients self-administer at home. Renal replacement therapy (RRT) means treatments which sustain life for patients with renal failure and includes all types of dialysis and transplantation.

## Methods

### Study design and setting

The study was designed as a mixed methods study: quantitative analysis of changes in home dialysis uptake rates over 3 years and qualitative case studies exploring factors that facilitated or impeded the desired change. The setting was at hospital renal units providing dialysis in the West Midlands and the United Kingdom. The design and analysis of the qualitative interviews was based on an intellectual framework. This was derived from two authoritative systematic reviews dealing, first, with the general topic of the diffusion and dissemination of complex health interventions [20], and second, the shifting of hospital services to community settings [21]. Factors identified from these two reviews were classified into four levels, using an established theoretical model for successful health system change [22]:

- Individual factors: how a service change operates with individual clinicians and patients;
- Team factors: how the clinical team is led, organised, trained, supported and funded;
- Organisational factors: how the strategy, culture and incentives of the wider organisation influence the service change;
- Wider system: how national and regional policies and commissioning influence the service change.

The resulting framework for the interviews was then cross-checked against national renal service guidance on home dialysis [8, 9], and a small number of extra items added to ensure maximum relevance to home dialysis. The framework included a statement for each factor setting out how it would be expected to be demonstrated within the renal service setting (Table 1).

**Table 1:** Framework for the qualitative interviews

Factors	Demonstrated by
Level 1: individual clinicians and patients [20, 21]	
Clinical pathway	A clear and up-to-date home dialysis pathway is in place and used by staff
Patient choice of treatment^a^	Patients are provided with timely and relevant information in a variety of formats to support their choice of treatment
	Staff promote home dialysis positively
Equipment^a^	There is an appropriate range of dialysis equipment available
	All staff have a good working knowledge of the dialysis equipment
	Patients can try out equipment before making a choice about treatment
	Property adaptations are timely
	Technical support, maintenance and adjustment to dialysis equipment is provided
Patient training and support^a^	High-quality patient training for home dialysis is provided using a variety of methods and techniques
	Peer support is available
	Ongoing support is provided to patients and carers
Patient feedback	Patient and carer feedback mechanisms are in place and are used by staff to adjust how they work
Level 2: renal team [20, 21]	
Vision	All staff share the vision and understand the home dialysis target
Leadership	There is visible and clear clinical and managerial leadership for home dialysis from trusted and influential individuals
	Leaders take personal responsibility, giving time to be involved and actively promote home dialysis
	Mechanisms are in place and used to overcome resistance to change
Staffing	Staffing competencies, grades and levels are consistent with the target
	The home dialysis target is reflected in job descriptions and appraisals
	Skill and training gaps are identified; training and development is put in place to address gaps
Culture	Staff have positive attitudes and support the target
	Staff at all levels are involved in planning and making changes to home dialysis, and their ideas and input are valued and used
	Innovation and change are actively promoted and staff are encouraged to try out new ideas for home dialysis in practice
Resources	Sufficient resources are available to meet the target (staff, equipment and funding)
Level 3: organisation (hospital) [20, 21]	
Strategy	The target contributes to the organisation’s current vision and strategy and is reflected in existing plans
	There is director-level sponsorship and senior leaders understand and actively promote the target
Incentives	Incentives for home dialysis are aligned with achievement
Level 4: wider NHS system [20, 21]	
Policy	National and regional policy supports the target
Commissioning	The commissioner’s strategy and contracting is aligned with the target
	The tariff and incentives/penalties are aligned with the target
Level 5: change management [20]	
Planning	A clear and realistic plan is in place for increasing uptake rates
	The baseline is mapped, and timely and accurate information is available to track progress
	Achievement against the plan is reviewed regularly, communicated to staff and adjusted when needed
Resources	Staff with the right skills and available time are leading the required changes

^a^Items added to the evidence-based framework from national renal policy documents

### Participants and data collection

Quantitative data was extracted from published Renal Registry reports for the calendar years 2007–2012 [16, 23–26], for the seven West Midlands hospitals (Dudley Group of Hospitals, Heart of England, Royal Shrewsbury and Telford, Royal Wolverhampton, University Hospitals Birmingham, University Hospitals Coventry and Warwickshire, University Hospitals North Staffordshire) and the 45 hospitals in the rest of England. This data is submitted by all hospitals in the United Kingdom, from a snapshot census of patients in treatment on 31st of December each year. The data is verified and cleansed before publication.

Four of the seven West Midlands hospitals were selected for in-depth qualitative case studies on the basis of achieving an urban/rural mix. Ethical approval was given by the University of Birmingham Research Ethics Committee (ERN 11-0479). Each hospital provided written R&D approval for the study. Each participant gave written consent to take part. Semi-structured qualitative interviews were conducted with patients and staff by experienced qualitative researchers (GC, KA, KS) with each researcher taking one-third of the randomly allocated interviews per hospital. Patients were eligible for the study if they had started their current dialysis treatment within the last 2 years and were aged 18+. They were excluded if surgery was scheduled in the next 3 months.

Each hospital provided the research team with an anonymised list of all the patients who met the selection criteria, along with details of each patient’s treatment type, age, sex and ethnicity. This was used to purposively sample by age (18–39, 40–64, 65+), sex, ethnic group and treatment type (PD, HHD, in-centre haemodialysis) in order to achieve diversity amongst the patients. Telephone interviews with 20–25 patients per hospitals were completed between November 2011 and March 2012. Interviews explored: how patients had come to be on dialysis, their experiences of each part of the dialysis pathway and their views about how to increase home dialysis uptake.

All staff groups who had contact with dialysis patients were eligible for interview. A potential list of interviewees was drawn up by the research team, based on interviewing: half of the consultants, at least one nurse from each of the specialist dialysis teams the wards and haemodialysis units and at least one of a list of specialist staff (see Table 2). The list was then discussed with the renal clinical lead to ensure all staff groups were covered. The majority of nursing staff were in senior/team leader roles. Face-to-face interviews were conducted between September 2011 and April 2012 with 20–30 staff per hospital. Interviews explored: current practice, using the last two or three patients seen by staff as exemplars, how well the dialysis pathway works, why patients do/do not opt for home dialysis and how the team had approached making the changes required to meet the home dialysis target. Hospitals also provided relevant documents for analysis.

**Table 2:** Roles of staff interviewed

Staff job role	Hospitals					
	1	2	3	4	Total	Total (%)
Renal consultant lead	1	1	1	1	4	
Renal consultant	8	6	3	2	19	
Clinical specialist	–	–	–	1	1	
Specialist registrar	2	2	1	–	5	
Sub-total doctors	11	9	5	4	29	30
Acute ward nurse manager	2	1	1	1	5	
Dialysis unit nurse manager	3	3	4	3	13	
Lead renal nurse/renal matron	1	–	–	1	2	
Pre-dialysis nurse/sister	1	1	3	1	6	
PD nurse/sister	2	–	–	2	4	
Home therapy nurse	–	4	3	–	7	
Home haemodialysis nurse/sister	2	–	–	2	4	
Sub-total nurses	11	9	11	10	41	43
Home therapy support worker	–	1	–	–	1	
Renal technician	1	1	1	1	4	
Psychologist	–	–	–	–	0	
Dietitian	1	1	–	1	3	
Consultant vascular surgeon	–	1	1	–	2	
Renal social worker/assistant	1	–	–	1	2	
Renal business manager	1	–	1	1	3	
Sub-total other renal staff	4	4	3	4	15	16
Hospital general managers	2	1	–	1	4	
Hospital clinical/medical director	1	2	1	1	5	
Hospital finance manager	1	–	–	1	2	
Sub-total hospital managers	4	3	1	3	11	11
Total	30	25	20	21	96	
Kidney Patients Association chair	1	–	–	1	2	
Number of interviews declined	3	0	7	0	10	

### Data analysis

The qualitative analysis was designed to look for explanations for the quantitative findings, particularly the mechanisms by which the target and financial penalty operated and the degree to which other factors also influenced uptake rates. The qualitative interviews were also designed to feed into the quantitative analysis by identifying background factors, such as population changes or changes in clinical practice, which might contribute to explaining changes in uptake rates, which could then be controlled for in the statistical analysis.

The primary quantitative outcome measure was the proportion of dialysis patients on home dialysis. The change in uptake rates between 2007 and 2012 for the West Midlands was compared with the rest of England. The numbers on home dialysis were analysed using a segmented logistic regression approach, with the break-point occurring between 2009 and 2010—the years just before and just after the introduction of the target. The model incorporates fixed effects for hospitals and separate linear time effects for the West Midlands and the rest of England. Overlap between eligible patient groups in consecutive years can be expected to generate temporal correlations within individual hospitals. Allowance for this effect was made through autoregressive models fitted using generalised estimating equations within the Stata 13 package. The analyses were adjusted for confounding variables which are known to affect dialysis uptake, including ethnicity and the proportion of the RRT population with transplants. The proportion of RRT patients aged under 65 was available only for the years 2009 onwards and was included in a separate analysis for these years. Comparison of four hospitals in the qualitative study with the three other West Midlands hospitals was also undertaken to look for a possible effect of being in the qualitative study (arising from selection bias and/or the Hawthorn effect).

Qualitative interview transcripts were coded by GC, KS and KA using fields derived from the evaluation framework, with 10 % of transcripts checked by a second researcher. Systematic analysis identified initial themes which were refined in team meetings and through further in-depth analysis. Themes were triangulated across the staff and patient interviews. Findings for each hospital were tested out and discussed with clinical staff at individual feedback meetings. Findings from the four hospitals were then triangulated and synthesised into a final report.

## Results

The full results of this study are available in a study report [27]. Here, we report on the findings relevant to the impact of the target and the issue of shifting from hospital care to home-based self-care for patients with complex medical needs.

### Quantitative results

**Fig. 1:**
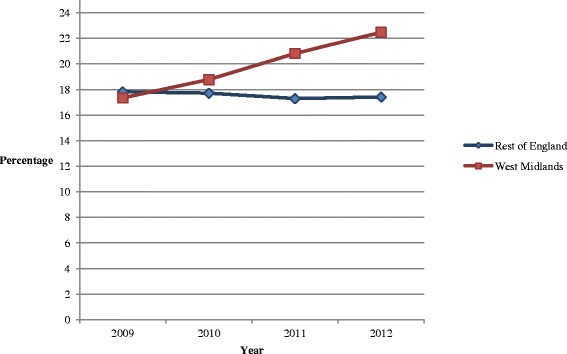
Percentage of dialysis patients on home dialysis

There was a fall in the proportion of dialysis patients using home dialysis across the whole of England in the 3 years pre-dating the target, from 20.6 % in 2007 to 17.9 % in 2009, with no significant difference between the rates of decline in the West Midlands and the rest of England (effect ratio 1.03, *p* = 0.546, Table 3). In the 3 years following the introduction of the target, the proportion rose in every West Midlands hospital to an average of 22.7 %; this contrasts with a slight fall from 18.0 to 17.4 % for the rest of England (Fig. 1, Table 4). The results of the logistic regression indicated that the year-on-year increases in the West Midlands between 2010 and 2012 were statistically significant (unadjusted odds ratio 1.15, *p* < 0.001) compared with a stable pattern for the rest of England (unadjusted odds ratio 1.00, *p* = 0.934). The effect ratio was significant in both adjusted and unadjusted models (ratio = 1.15, *p* < 0.001, Table 3).

**Table 3:** Segmented logistic regression analysis of rates of home dialysis per dialysed patient, 2007–2012

	Odds ratio	Confidence interval	*P* value
Unadjusted analysis			
Time effects (per year)			
2007 to 2009: West Midlands	0.94	(0.87, 1.01)	0.085
Rest of England	0.91	(0.88, 0.95)	<0.001
Ratio (W.Mids:Rest)	1.03	(0.94, 1.11)	0.546
2010 to 2012: West Midlands	1.15	(1.07, 1.23)	< 0.001
Rest of England	1.00	(0.97, 1.03)	0.934
Ratio (W.Mids:Rest)	1.15	(1.06, 1.24)	< 0.001
Adjusted analysis			
% RRT patients aged under 65^a^	1.00	(0.98, 1.03)	0.598
% RRT patients transplanted	1.02	(1.00, 1.03)	0.041
% RRT patients from BME groups	1.00	(0.98, 1.02)	0.898
Time effects (per year)			
2007 to 2009: West Midlands	0.94	(0.86, 1.01)	0.103
Rest of England	0.90	(0.87, 0.93)	< 0.001
Ratio (W.Mids:Rest)	1.04	(0.95, 1.13)	0.385
2010 to 2012: West Midlands	1.14	(1.06, 1.22)	< 0.001
Rest of England	0.99	(0.96, 1.02)	0.433
Ratio (W.Mids:Rest)	1.15	(1.07, 1.25)	< 0.001

^*^Ages unavailable before 2009. Age-effect estimated from separate analysis using data from 2009 to 2012 only

**Table 4:** Changes in the RRT population and the proportion of dialysis patients on home dialysis, 2009–2012

Region	RRT Population	Year					
		2007	2008	2009	2010	2011	2012
West Midlands (7 Trusts)	Total RRT population	4490	4740	4983	5113	5315	5434
	% patients transplanted	38.3	38.4	38.2	39.0	39.7	40.0
	% patients under 65*	–	–	62.6	62.6	62.4	62.1
	% patients from BME groups	23.2	23.7	24.0	24.7	25.1	25.4
	Total on dialysis	2769	2922	3078	3120	3204	3259
	Number of on-home dialysis	552	538	534	586	667	740
	% dialysis patients at home	19.9	18.4	17.3	18.8	20.8	22.7
Rest of England (45 Trusts)	Total RRT population	33124	34736	35979	37547	39350	40642
	% patients transplanted	47.8	48.2	48.7	49.8	50.6	51.4
	% patients under 65^a^	–	–	66.4	66.0	64.9	64.2
	% patients from BME groups	19.1	20.1	20.5	21.2	21.7	22.2
	Total on dialysis	17277	17991	18466	18858	19450	19734
	Number of on-home dialysis	3576	3468	3330	3356	3359	3438
	% dialysis patients at home	20.7	19.3	18.0	17.8	17.3	17.4

^a^Data not available

Source: Renal Registry Annual Reports [15, 23–26]

The qualitative analysis suggested that changes in population characteristics during the study period, particularly age and ethnicity, might have influenced uptake rates, and these are summarised in Table 4. The total number of RRT patients and the percentage transplanted rose in the West Midlands broadly in line with national trends but with rates remaining lower than national averages. The proportion of black and minority ethnic (BME) RRT patients rose in line with national trends and remained above that for the rest of England. The proportion of RRT patients aged 65+ remained static in the West Midlands after 2009 whilst national rates fell slightly. Neither the proportion under 65 (odds ratio 1.00, confidence interval (CI) 0.98 to 1.02) nor the proportion of BME patients (odds ratio 1.00, CI 0.98 to 1.02) contributed significantly to the trends in home dialysis uptake. The effect of changes in the percentage of transplanted patients was marginally significant at the 5 % level in the adjusted model, in the direction of a slight increase in the home dialysis rate as the proportion of transplants increased (odds ratio 1.02, CI 1.00 to 1.03, *p* = 0.041).

In a separate analysis, the ratio of post 2009 time-effects between the four hospitals in the qualitative study and the remaining West Midlands hospitals was 1.01 (CI 0.92 to 1.10, *p* = 0.899). Thus, there was no evidence for a selection or Hawthorn effect. Data on the annual interim targets and whether they were met were considered by Trusts to be commercially confidential and were not therefore made available to the research team.

### Qualitative results

Of the 618 eligible patients across the four hospitals, 101 (16 %) were contacted to take part and 93 were interviewed (21–25 per hospital), with 8 refusals (Table 5). Table 6 summarises the demographic features of the eligible and sampled patients. The sampling strategy was amended during fieldwork in the first hospital to include patients starting treatment within the last 24 months, rather than 12 months, due to the small number of eligible patients in certain categories. There were no observed effects from this change on data quality, particularly on patients’ abilities to recall details of their treatment and decision-making. One hundred and six staff were invited to take part in the study, and 10 (9 %) refused to take part, resulting in a sample size of 96 (20–30 per hospital). Table 2 summarises the roles of the staff who were interviewed. There were no withdrawals of patients or staff from the study.

**Table 5:** Patient sampling, case studies

Patient sample	Hospitals				Total
	1	2	3	4	
Eligible	205	152	129	132	618
Refusals	**–**	5	3	0	8
Interviewed	23	25	21	24	93
Eligible patients interviewed (%)	11	16	16	18	15

**Table 6:** Patient characteristics, case studies

Patient characteristics	Hospitals				Total	Percentage	No. of eligible patients	Eligible patients interviewed (%)
	1	2	3	4				
Treatment type								
PD	10	11	11	8	40	43	181	22
Home haemodialysis	4	7	1	6	18	19	28	64
In-centre haemodialysis	9	7	9	10	35	38	409	9
Sex^a^								
Male	14	18	12	11	55	59	359	15
Female	9	7	9	13	38	41	230	17
Age group								
18–39	5	5	3	5	18	19	67	27
40–64	13	8	8	9	38	41	223	17
65+	5	12	10	10	37	40	328	11
Ethnic group^a^								
White	13	25	15	23	76	82	509	15
Indian	6	0	2	1	9	10	52	17
Pakistani	2	0	0	0	2	2	23	9
African Caribbean	2	0	4	0	6	6	33	18

^a^Missing data: sex not recorded for 29 eligible patients not included in the study; ethnic group not recorded for 10 eligible patients not included in the study

Table 7 summarises the main actions taken by the hospitals in order to increase home dialysis uptake rates. There was no one set or subset of actions which was clearly associated with higher or lower uptake rates in individual hospitals. Instead, we identified a number of barriers and facilitators which were common to all hospitals.

**Table 7:** Summary of actions taken by hospitals to increase the uptake of home dialysis

Actions taken	Hospitals			
	1	2	3	4
Resources				
Significant additional resources secured from the hospital for staff and home dialysis machines	✓	✓	✓	✓
Forward-looking resource and capacity plan developed for achieving the 2015 target for home haemodialysis	✓			
Widening access				
Assisted PD introduced to widen access to more frail patients or those living alone	✓	✓	✓	✓
Rapid/direct access to PD for acute patients to prevent acute patients automatically going onto in-centre haemodialysis	✓	✓	✓	✓
Rapid PD catheter insertion	✓			✓
Solo home haemodialysis introduced, so patients do not need to have a carer involved				✓
Portable home haemodialysis machine introduced				✓
Self-care/minimal care routinely available in in-centre haemodialysis units as a possible stepping stone to home haemodialysis				✓
One-off reviews of in-centre haemodialysis patients’ treatment options		✓	✓	✓
In-centre haemodialysis patients successfully switched to home dialysis				✓
Peer support				
Peer support scheme for patients interested in home haemodialysis	✓			
Informal peer support available for patients interested in home dialysis		✓		
Staffing, training and induction				
Home dialysis included in the induction of all new staff	✓			✓
Staff rotation used to increase staff knowledge of home dialysis		✓		✓
Hospital support				
Visible support secured from hospital senior management	✓			✓
Home dialysis targets deliberately aligned with the hospital’s strategic plan	✓	✓		✓
Approach to the target				
Focus on increasing both home haemodialysis and PD uptake		✓	✓	✓
Focus solely on increasing home haemodialysis	✓			

#### Facilitators

The primary facilitator in all four hospitals was the commissioner’s target and financial penalty. However, this was found not to be a sufficient explanation for the observed changes. Three additional facilitators were also operating: new funding for specialist staff and dialysis machines, clinical leadership and wider staff support and the training and support systems for home dialysis patients. The facilitators are explored in turn (Table 8).

**Table 8:** Main facilitators in all hospitals

Commissioner’s target and financial penalty scheme
“You know and it’s always a cost issue isn’t it? No matter what, patient care is cost, that’s what it is isn’t it? And that, I think that’s wrong.” Nurse, hospital 3, February 2012
“I'm slightly wary of targets, that to achieve a target we could be pushing it to people who aren't happy with it.” Consultant, hospital 1, November 2011
Funding for additional specialist staff and dialysis machines
“But also the commissioners, by having a bit of a stick as well as a carrot for us to achieve higher home therapy rates, [it] has been very helpful in our negotiations with our Trust [hospital] to say “look, we’ll lose this amount of money if we don’t invest to achieve it”.” Clinical lead, hospital 4, March 2012
Clinical leadership and wider staff support
“I think we’re fortunate to have staff who want to do this ..... it’s been driven by enthusiastic staff wanting to provide, you know, better care for their patients.” Centre clinical lead, medicine, hospital 4, April 2012 “I’m liking the way now it’s [home dialysis] coming back in to the fore again. Because I think it is so much better for the patients than having to get on transport, taken all round the area before they come here and then waiting for transport again.” Haemodialysis unit nurse manager, hospital 2, October 2011
Training and support systems for home dialysis patients
“….they’ll say some patients need 3 days [training], some patients need 7, some people need 2 weeks. So we go as quick as what you need to go. So its quite good really.” PD Patient, (9) hospital 1, November 2011

##### The commissioner’s target and financial penalty scheme

There was clear evidence that the commissioner’s target had acted as a strong incentive for hospitals to increase the uptake of home dialysis and that this had been the most important facilitator. This was in part due to the significant financial penalty which was incurred if interim annual targets were missed which were equivalent to around 1 % of total renalincome. Many staff reported having negative reactions to the target when it was introduced in 2010, because of perceptions of insufficient consultation about how the target was set, concerns that the target might subtly influence clinical judgement and criticisms that the target was not evidence-based. Despite these initial negative reactions, at the time of interviewing, nearly all staff thought the target had worked well in getting staff to focus on increasing the uptake of home dialysis and that interim annual targets had kept staff focussed and helped progress to be made year-on-year. Overall, there was good qualitative evidence that the target plus financial penalty had acted as a strong incentive and had directly resulted in uptake rates increasing at a speed which would not have been achieved otherwise.

##### Funding for additional specialist staff and dialysis machines

Hospitals were clear that they had needed additional funding for staff and dialysis machines in order to increase uptake rates, but this was not forthcoming from the commissioner. The renal clinical leads had all used the target to argue successfully for significant additional resources from their organisations. In three hospitals, new home therapy nursing posts had been agreed within the previous 12–24 months, along with additional surgical capacity. In two hospitals, new consultant posts were funded and in another hospital a new technician post was funded. Two different approaches to securing additional resources were evident. In hospital 1, there was a business plan setting out the additional staff capacity required to enable delivery of the home haemodialysis target, which resulted in new posts being funded. In contrast, hospital 4 had had to demonstrate increases in home haemodialysis uptake which caused workload pressures *before* additional posts were funded.

##### Clinical leadership and wider staff support

Strong clinical leadership was seen by staff as key to success in increasing home dialysis uptake rates, with particular individuals being highlighted as visible and effective champions. Support from renal clinical leads was also seen as essential in creating the right climate for change. A combination of strong clinical leaders, individual champions for home dialysis and enthusiastic home therapy nursing teams was frequently identified as important. Also notable was the breadth of support for home dialysis amongst senior renal staff on the acute wards and the haemodialysis units. These staff were all aware of and expressed support for increasing uptake rates.

##### Training and support systems for home dialysis patients

Feedback from staff and patients suggested that the training and support systems were working very well. There were only minor improvements suggested by patients who tended to be fulsome in their praise—training was seen as timely, well organised and relevant, preparing patients very well for home dialysis. Ongoing support via the telephone or through home visits and out-patient appointments also worked well for staff and nearly all patients. There were no suggestions that any significant changes needed to be made, although some staff thought the systems for ongoing support might become stretched if uptake rates continued to rise.

#### Barriers

Barriers related to housing, space at home and the ordering and installation of dialysis machines had been anticipated from pre-study discussions with hospitals but were not found. Just one hospital reported difficulties in ordering home haemodialysis machines, but this supply chain issue was quickly resolved. Three barriers were found in all hospitals: lack of training for non-specialist staff, pre-dialysis education and a lack of recognition by staff of the patients’ emotional and psychological needs. These are explored in turn (Table 9).

**Table 9:** Barriers

Lack of training for non-specialist staff
“None [time spent on training about home therapies]. I very rarely get involved with PD peritonitis but that’s about it, nothing else and nothing on home haemodialysis.” Specialist Registrar, hospital 3, January 2012
“....it was actually one of the health care assistants, I was asking her about something to do with the [haemodialysis]machine and she said “Oh I don’t know what you’re bothered about asking for, you’re not going home…” and I was completely if you like shot down in flames over it. And I’m like I’m asking questions because I’m interested..... I mean for some people they’d just go “OK I won’t bother asking then”.” Home haemodialysis Patient, (24) hospital 4, March 2012
Pre-dialysis education
“Speaking directly to someone who has had it [dialysis], so you’re getting all the unfiltered information…it was useful to be able to speak to a person who had gone through that to give us, you know, warts and all what’s going to happen…” PD Patient, (15) hospital 4, March 2012
Patients’ unmet psychological and emotional needs
“I went through a period towards the end of my preparations for dialysis where I had to go to the doctor with depression because I was just so unhappy because I felt sick every day and my whole life just kind of crumbled around me really.” Home haemodialysis Patient (20), hospital 4, March 2012
“So they focus totally on the practical side of things. Have they done it? Why haven’t they done it? You’re going to die if you don’t do it… No disrespect, but sometime you don’t want to tell them you’ve got a problem… [There’s] a huge mental side to it, well I don’t know what you’d call it, a psychological element they probably don’t quite press.” PD Patient, (4) hospital 3, February 2012
“I have to admit for the first 12 months or so I found it very, very depressing. I couldn’t get my head round it, with these big bloody needles going up my arm, maybe for the next 10 years or so.” In-centre haemodialysis Patient, (9) hospital 3, February 2012
“So quite often people are shocked, you know, they just kind of don’t know what to think really about anything…. I kind of equate it to like the grieving, really they’ve kind of lost their kidneys and it’s almost like a death for them… they kind of go through all those emotions that come with bereavement.” Dialysis unit nurse manager, hospital 4, March 2012
“Some patients need listening to when they’re not well, you know because a lot of them suffer from depression, they get stuck in a rut sometimes, they just need 5 minutes to explain how they’re feeling about their illness”. Home haemodialysis Patient,7) hospital 2, November 2011

##### Lack of training for non-specialist staff

Renal staff working on the wards and in haemodialysis units said they lacked confidence in talking with patients about home dialysis, even on a casual basis. With the exception of ward staff who provided out of hours support to PD patients, most staff had had no recent training about home dialysis. Hospital 4 was the exception, where all staff were well informed and felt confident in talking to patients about home dialysis. This was the only hospital which used induction and training programmes to ensure *all* renal staff knew the basics about home dialysis. Although staff wanted training, they also wanted to see patients treating themselves at home, because this was seen as the most effective learning method. Specialist registrars in particular highlighted that they were having conversations regularly with patients about treatment options, despite having spent very little time on home dialysis in their training programme.

The importance of training for *all* staff was reinforced by the patient interviews. Patients said their casual conversations with staff about treatment options and their questions were often not dealt with well by staff on the wards and in the haemodialysis units. Staff often failed to portray the benefits of home dialysis positively and had missed opportunities to encourage patients to consider home dialysis.

##### Pre-dialysis education

Pre-dialysis education was designed to support patients in choosing the right dialysis treatment for them. This involved offering patients one-to-one sessions about treatment options with specialist nursing staff, and the opportunity to attend group sessions, which usually included talks by patients on dialysis. Both staff and patients thought many patients found treatment choice very difficult because of the number of treatment options and complexity of information. Some patients described having “information overload”. They wanted a wider range of teaching methods to be used, including active methods which would allow patients to handle dialysis equipment and see treatment in action. These were seen as ways of making the treatment options “real” to patients.

Staff and patients seemed to have very different views about how patients make treatment decisions. Staff tended to describe a rational weighing of treatment options which was based largely on information. In contrast, most patients described a more personalised approach of thinking about their own lives and how different options might work for them. Although information was important, its application to patients’ own lives was more significant. Some patients described one main reason for their choice of treatment, whilst others could not articulate why they had opted for their treatment. None of the patients who were interviewed had been offered peer contact or support as a formal part of the pathway, although it had recently been introduced in hospital 1 for patients interested in home haemodialysis, and hospital 2 would put pre-dialysis patients in touch with established patients in response to patient requests. It was notable that the most common suggestion from patients for improving the service was to have more opportunities to talk to and be supported by other patients.

Some patients described a gradual process of decision-making and thought they had benefited from being able to make their treatment choice over a period of time. However, there were other patients who had felt unable to make a choice, despite having known they would need dialysis for years. Some described how their strong emotional reactions to reaching end-stage renal failure had left them unable to make decisions or too scared to consider home dialysis. These patients talked about becoming interested in home dialysis only once they had started dialysis themselves, and it was thus significant that none of the hospitals had built routine reviews of treatment choice into their dialysis pathways.

##### Patients’ unmet psychological and emotional needs

The most striking and significant barrier this study uncovered was that many patients had found it hard to adjust psychologically and emotionally to the need for dialysis, but that this was not well recognised or responded to by staff. Although this was not something the study had set out to explore, just over a third of patients talked openly about the transition to dialysis as a scary and traumatic experience. Most of these patients had established chronic kidney disease and had known they would need dialysis years in advance of starting dialysis. Despite this, they still described feelings of shock and trauma when it became clear they would need dialysis soon. Some patients talked at length about becoming depressed and feeling isolated, with distress continuing even when they were well established on dialysis.

In contrast, there appeared to be an almost complete absence of service responses to patients’ distress and its impact on their ability and confidence to choose home dialysis. Just three staff mentioned patients’ emotional and psychological needs as significant. None of the hospitals had adapted their pre-dialysis pathways or training processes to take account of patients’ distress, and none had support arrangements in place for patients other than referral to a psychiatrist/psychologist for depression. Patients said they wanted staff to ask about the wider impact of dialysis on their lives and not to focus solely on the medical aspects of their illness. They wanted opportunities to talk and be listened to.

## Discussion

This study provides insight into how renal services can increase the uptake of home dialysis and identifies the facilitators and barriers to doing this within a relatively short period of time. It is also an interesting case study of the impact of a target and for how care can be re-provided from professional-led hospital care to home-based self-care for patients with complex medical needs.

### Pay-for-performance

The findings from this study are in line with the literature on the use of targets with financial penalties/rewards in a number of ways. Firstly, systematic review evidence suggests that using pay-for-performance to achieve pre-specified changes in activity, as in this study, is one of the most effective uses of targets and tends to achieve positive results [19]. Secondly, the finding of a 5 % greater home dialysis treatment uptake rate in the study hospitals compared with the rest of England is within the positive effect sizes of 1–10 % identified by a recent systematic review of pay-for-performance schemes [17] and the 2–4 % improvements found in the single biggest US hospitals study [28]. As the only known factor distinguishing the hospitals in the current study was the target and as the qualitative study confirmed that this had stimulated changes designed to increase uptake rates, it is concluded that the target played a key role in changing home treatment uptake rates. Interestingly, the literature suggests that positive effects are most likely to be found where the room for improvement is the greatest [17, 18]. This applies to home dialysis treatment uptake rates in England which have been well below the potential level suggested by the evidence base (see “Background” section).

Thirdly, the qualitative part of the study identified some factors facilitating increased home treatment uptake which are in line with the literature on the provider characteristics associated with positive outcomes from the use of targets: effective clinical leadership [17, 18]; ownership of the target at team level [17, 18]; a multidisciplinary team approach [29]; and having sufficient staff and funding to effect change [29]. It also worth noting that several of the design features of the target in the study hospitals have been identified in the literature as problematic and unlikely to lead to positive outcomes, notably: rewarding performance by re-allocating existing funds rather than providing new funding and not involving providers in selecting and setting targets and rewards/penalties [17, 18]. This suggests there could be the potential for even greater increases in the uptake of home dialysis treatments in the future if these design features were avoided.

Moving on to the broader implications, this study suggests that four important service elements need to be in place if patients with complex medical needs are to be encouraged to opt for home-based self-care. First, information, guidance and support for patients, to help them make a realistic decision about whether to have hospital or home dialysis. Second, for patients opting for home dialysis, high-quality training in the use of dialysis machines so that they are competent to self-care at home. Third, ongoing technical assistance and support for patients once they are on home dialysis. Fourth, emotional and psychological support designed to help patients adjust to end-stage renal failure. Interestingly, we found that only two of these four service elements were working well. Both the training and ongoing support systems were very well developed. Patient feedback highlighted training as exemplary, with patients valuing the fact that it was adapted to meet individual patients’ needs, and lasted as long as was needed to develop patient competence and confidence. In a similar vein, technical support was provided 24/7 for patients at home, along with regular home visits by specialist nurses and telephone support on request.

In contrast, emotional and psychological support for patients was poorly developed, with little recognition amongst staff of the scale of the need. Supportive emotional care is important for two reasons. First, it helps patients during a difficult transition in treatment, potentially reducing depression and improving a sense of well-being. Second, patients must be supported emotionally if they are to think through a difficult treatment choice and seriously consider taking on the undoubted challenges of home-based self-care.

### Improving emotional well-being

The level of need, with over a third of our study patients reporting experiencing distress during their transition to end-stage renal failure and dialysis, is similar to accounts in the literature [30–32]. Although only a few of our study patients reported being treated for depression, many were in considerable distress. The observed absence of support for patients in emotional distress which falls below the threshold of clinical depression is in keeping with the literature [33–35]. This is despite evidence that such levels of emotional and psychological distress are associated with lower life expectancy, increased hospitalisation and poorer treatment adherence amongst renal patients [36].

### Supporting decision-making

The information, guidance and support to help patients make a decision about treatment options were also poorly developed and feedback from patients was clear. They wanted less of a focus on technical information and more support to help them apply information to their own lives. The literature suggests that high stress levels in the pre-dialysis period are a significant predictor for choosing hospital rather than home dialysis [37]. Systematic review evidence suggests that self-care choices are more likely to be made by patients when physicians have individualised conversations with patients about what is important to them, what they value and hindrances to self-care [38]. Consistent with these findings from the literature, our study found that some patients said that their distress had impeded their decision-making and prevented them from giving full consideration to home dialysis.

### Strengths and limitations

To our knowledge, this is the first multi-site evaluation of how hospitals have increased the uptake of home dialysis. One of its strengths is that the hospitals started working on the same target at the same time and that reliable comparative data was available to track the impact on uptake rates. The relatively large sample size in the qualitative study lends weight to the findings, as does the use of a purposive sample designed to capture diverse patient views and experiences.

The study had some limitations. The qualitative study hospitals were not selected to be representative of renal services across the country or the region. However, the similarity of results across hospitals suggests a degree of generalisability. The snapshot nature of the quantitative data is potentially limiting, although there is no evidence for seasonal variations in uptake rates for any type of RRT. The single point in time for qualitative data collection provides less insight than multiple data collection points over time. Sampling may have introduced some bias as we were unable to recruit the planned number of patients in some categories of the purposive sample. The fact that majority of nursing staff interviewed were in leadership roles could have led to some bias in favour of home treatment, as frontline staff might be expected to be less aware of and engaged in meeting external targets.

### Further research

Research is needed to identify and evaluate ways of meeting patients’ emotional and psychological needs during transitions in patients’ illnesses. We suggest that clinical staff are in the best position to support patients through difficult transitions in their illnesses because they have ready-formed relationships. Research should therefore focus on how emotional needs can be discussed during routine appointments with doctors and how specialist nurses can incorporate emotional support into their discussions about treatment options, particularly when patients are going through a significant transition in their illness. We believe this is a very important area of research which would be of wider relevance to services for patients with various life-threatening long-term conditions.

## Conclusions

This study showed the power of a target with a financial penalty to stimulate renal services to increase the uptake of home dialysis, something which had not been achieved previously despite favourable policies and evidence. We conclude that without the target, this change would not have occurred across the seven study hospitals but also that a number of additional barriers and facilitators influenced the change in uptake rates. Although this is an interesting case study within renal services, we believe that many of the issues are relevant to services for patients with other complex and life-threatening conditions. In particular, the twin issues of emotional support and support for treatment decision-making are highly relevant across other services. As the number of treatment options for patients with complex medical needs increases, and with more emphasis on patient choice, the issue we highlight of how best to support patient decision-making in a non-directive way will become increasingly important. Based on our findings, we suggest that the current focus on providing detailed complex information to patients and leaving them to make a choice needs to change. Patients need a more individualised approach which helps them to express their feelings about treatment options and think through what each treatment would mean in practice in their own lives. Without this, it seems unlikely that patients will opt for home-based self-care in large numbers. For many patients, it will seem too demanding, requiring new skills and a degree of courage to use technical equipment without the presence of healthcare professionals. We acknowledge that this kind of exploration of treatment options with patients is also demanding for health professionals and will require the development of specialist skills which are more akin to counselling than patient education.

Finally, we suggest that the lack of recognition of the role of healthcare professionals in providing emotional and psychological support to all patients, as found in our study, is one of the most significant barriers to shifting healthcare from hospital settings to self-care at home for patients with complex medical needs. It seems unlikely that self-care at home will take off, except amongst the most resourceful, educated and resilient patients, without the routine provision of emotional and psychological support to patients and the upskilling of healthcare professionals to recognise and respond to these needs.
